# Supplementary material for: First comprehensive analysis of Aedes aegypti bionomics during an arbovirus outbreak in west Africa: Dengue in Ouagadougou, Burkina Faso, 2016–2017
Source: PLoS Negl Trop Dis. 2022 Jul 6;16(7):e0010059. doi: 10.1371/journal.pntd.0010059 (PMC9321428; doi:10.1371/journal.pntd.0010059)
Supplement: S1 Table — (DOCX) [file pntd.0010059.s001.docx]

**S1Table**. Household characteristics and demography in the three localities during the two years of collection, 2016 and 2017.

|  |  | 1200 Logements | | | | | | | |  | | Tabtenga | | | | | |
| --- | --- | --- | --- | --- | --- | --- | --- | --- | --- | --- | --- | --- | --- | --- | --- | --- | --- |
| STATISTIC |  | | Aug-16 | Sep-16 | Oct-16 | Aug-17 | Sep-17 | Oct-17 |  | | Aug-16 | | Sep-16 | Oct-16 | Aug-17 | Sep-17 | Oct-17 |
| Premises visited |  | | 114 | 115 | 158 | 49 | 53 | 41 |  |  | 129 | | 184 | 81 | 101 | 100 | 91 |
| Number of residents |  | | 635 | 621 | 748 | 288 | 230 | 205 |  |  | 556 | | 944 | 360 | 520 | 405 | 353 |
| Premises positive for adult *Ae. aegypti* |  | | 93  (82.3%) | 104  (89.7) | 105  (66.5%) | 41  (83.7%) | 43  (79.6%) | 24  (58.5%) |  |  | 84  (66.1%) | | 128  (69.2%) | 36  (44.4%) | 77  (77.0%) | 72  (71.3%) | 36  (39.1%) |
| Premises positive for pupae and larvae (HI) |  | | 67 (58.8%) | 54 (47.4%) | 19 (16.7%) | 26 (22.8%) | 20 (17.5%) | 0 (0.0%) |  |  | 66 (57.9%) | | 69 (60.5%) | 7 (6.1%) | 51 (44.7%) | 38 (33.3%) | 10 (8.8%) |
| Premises positive for pupae (HPI) |  | | 49 (43.0%) | 44 (38.3%) | 10 (6.3%) | 21 (42.9%) | 11 (20.8%) | 0 (0.0%) |  |  | 40 (31.0%) | | 56 (30.4%) | 5 (6.2%) | 40 (39.6%) | 25 (25.0%) | 2 (2.2%) |
| No. containers with Water |  | | 167 | 144 | 58 | 114 | 61 | 0 |  |  | 197 | | 218 | 17 | 196 | 85 | 20 |
| No. containers positive for pupae and larvae (CI) |  | | 94 (56.3%) | 101 (70.1%) | 27 (46.6%) | 71 (62.3%) | 43 (70.5%) | 0 (-) |  |  | 82 (41.6%) | | 110 (50.5%) | 7 (41.2%) | 80 (40.8%) | 49 (57.7%) | 7 (35.0%) |
| No. containers positive for pupae (CPI) |  | | 65 (38.9%) | 64 (44.4%) | 11 (0.19) | 50 (43.9%) | 22 (36.1%) | 0 (-) |  |  | 47 (23.9%) | | 79 (36.2%) | 5 (29.4%) | 55 (28.1%) | 33 (38.8%) | 2 (10.0%) |
| Total number of pupae |  | | 690 | 965 | 99 | 616 | 202 | 0 |  |  | 897 | | 1625 | 52 | 927 | 695 | 16 |
| Pupae per person index |  | | 1.1 | 1.6 | 0.1 | 2.1 | 0.9 | 0.0 |  |  | 1.6 | | 1.7 | 0.1 | 1.8 | 1.7 | 0.0 |
| Breteau Index |  | 48.0 | | 51.5 | 13.8 | 36.2 | 21.9 | 0.0 | |  | | 41.8 | 56.1 | 3.6 | 40.8 | 25.0 | 3.6 |

|  |  | Goundry | | | | | |
| --- | --- | --- | --- | --- | --- | --- | --- |
| STATISTIC |  | Aug-16 | Sep-16 | Oct-16 | Aug-17 | Sep-17 | Oct-17 |
|  |  |  |  |  |  |  |  |
| Premises visited |  | 196 | 146 | 47 | 53 | 68 | 54 |
| Number of residents |  | 548 | 393 | 127 | 137 | 222 | 213 |
| Premises positive for adult *Ae. aegypti* |  | 38  (18.9%) | 27  (18.5%) | 3  (6.4%) | 18  (34.0%) | 22  (32.8%) | 10  (18.5%) |
| Premises positive for pupae and larvae (HI) |  | 86 (75.4%) | 44 (38.6%) | 8 (7.0%) | 33 (29.0%) | 24 (21.1%) | 11 (9.7%) |
| Premises positive for pupae (HPI) |  | 46 (23.5%) | 25 (17.1%) | 3 (6.4%) | 20 (37.8%) | 11 (16.2%) | 8 (14.8%) |
| No. containers with Water |  | 428 | 242 | 22 | 182 | 132 | 35 |
| No. containers positive for pupae and larvae (CI) |  | 151 (35.3%) | 68 (28.1%) | 10 (45.5%) | 71 (39.0%) | 37 (28.0%) | 16 (45.7%) |
| No. containers positive for pupae (CPI) |  | 55 (12.9%) | 26 (10.7%) | 1 (4.6%) | 34 (18.7%) | 12 (9.1%) | 10 (28.6%) |
| Total number of pupae |  | 1522 | 468 | 35 | 990 | 145 | 160 |
| Pupae per person index |  | 2.8 | 1.2 | 0.3 | 7.2 | 0.7 | 0.8 |
| Breteau Index |  | 77.0 | 34.7 | 5.1 | 36.2 | 18.9 | 8.2 |
