# Supplementary material for: First comprehensive analysis of Aedes aegypti bionomics during an arbovirus outbreak in west Africa: Dengue in Ouagadougou, Burkina Faso, 2016–2017
Source: PLoS Negl Trop Dis. 2022 Jul 6;16(7):e0010059. doi: 10.1371/journal.pntd.0010059 (PMC9321428; doi:10.1371/journal.pntd.0010059)
Supplement: S2 Table — (DOCX) [file pntd.0010059.s002.docx]

**S2Table**. Average number per house, the 95% confidence limits [in brackets] and the total number of mosquitoes (in parenthesis) of *Aedes aegypti* mosquitoes collected in the study localities in 2016 and 2017

| Location |  | Locality | | | | | | | |
| --- | --- | --- | --- | --- | --- | --- | --- | --- | --- |
|  |  | 1200LG (urban) | |  | Tabtenga (peri-urban) | |  | Goundry (rural) | |
|  |  | 2016 | 2017 |  | 2016 | 2017 |  | 2016 | 2017 |
| Indoor |  | 0.51(426)  [0.41-0.61] | 0.37 (123)  [0.25-0.49] |  | 0.48 (333)  [0.4-0.57] | 0.67 (371)  [0.54-0.81] |  | 0.04 (25)  [0.02-0.06] | 0.05 (17)  [0.02-0.09] |
| Outdoor |  | 1.79 (1,373)  [1.54-2.07] | 1.86 (659)  [1.46-2.33] |  | 0.83 (643)  [0.69-0.98] | 0.89 (592)  [0.71-1.07] |  | 0.16 (112)  [0.12-0.21] | 0.28 (126)  [0.19-0.39] |
