# Supplementary material for: First comprehensive analysis of Aedes aegypti bionomics during an arbovirus outbreak in west Africa: Dengue in Ouagadougou, Burkina Faso, 2016–2017
Source: PLoS Negl Trop Dis. 2022 Jul 6;16(7):e0010059. doi: 10.1371/journal.pntd.0010059 (PMC9321428; doi:10.1371/journal.pntd.0010059)
Supplement: S3 Table — (DOCX) [file pntd.0010059.s003.docx]

**S3Table.** Average number of mosquitoes per house, the 95% confidence limits [in brackets] and the total number (in parenthesis) collected in the morning (am) and in the afternoon (pm) of the three main species of mosquito collected in the study localities in 2016 and 2017.

| collection time | Locality | | | | | | | |
| --- | --- | --- | --- | --- | --- | --- | --- | --- |
|  | 1200LG (Urban) |  |  | Tabtenga (peri-urban) | |  | Goundry (Rural) | |
|  | 2016 | 2017 |  | 2016 | 2017 |  | 2016 | 2017 |
| am | 1.37 (1,123)  [1.15-1.61] | 1.27 (418)  [0.94-1.66] |  | 0.64 (572)  [0.54-0.75] | 0.86 (467)  [0.7-1.03] |  | 0.08 (49)  [0.04-0.11] | 0.1 (64)  [0.04-0.17] |
| pm | 0.8 (688)  [0.67-0.94] | 0.78 (364)  [0.59-1] |  | 0.63 (404)  [0.52-0.76] | 0.7 (496)  [0.56-0.85] |  | 0.11 (88)  [0.07-0.14] | 0.21 (79)  [0.14-0.29] |
