# Supplementary material for: First comprehensive analysis of Aedes aegypti bionomics during an arbovirus outbreak in west Africa: Dengue in Ouagadougou, Burkina Faso, 2016–2017
Source: PLoS Negl Trop Dis. 2022 Jul 6;16(7):e0010059. doi: 10.1371/journal.pntd.0010059 (PMC9321428; doi:10.1371/journal.pntd.0010059)
Supplement: S4 Table — (DOCX) [file pntd.0010059.s004.docx]

**S4Table**. Number of bloodfed, number of PCR-tested, and bloodmeal sources of *Aedes aegypti* mosquitoes per locality and per year.

| Year | Locality | Total collected | Total Tested | Human | | Dog | Human+Dog | Dog+  Cow |  |
| --- | --- | --- | --- | --- | --- | --- | --- | --- | --- |
| 2016 | 1200LG | 304 | 104 | | 55 | 2 | 2 | 0 |  |
|  | Tabtenga | 202 | 106 | | 61 | 1 | 3 | 0 |  |
|  | Goundry | 18 | 14 | | 5 | 1 | 0 | 1 |  |
| 2017 | 1200LG | 101 | 101 | | 27 | 9 | 0 | 0 |  |
|  | Tabtenga | 166 | 166 | | 10 | 3 | 0 | 0 |  |
|  | Rural | 7 | 7 | | 0 | 0 | 0 | 0 | |
