# Supplementary material for: First comprehensive analysis of Aedes aegypti bionomics during an arbovirus outbreak in west Africa: Dengue in Ouagadougou, Burkina Faso, 2016–2017
Source: PLoS Negl Trop Dis. 2022 Jul 6;16(7):e0010059. doi: 10.1371/journal.pntd.0010059 (PMC9321428; doi:10.1371/journal.pntd.0010059)
Supplement: S5 Table — (DOCX) [file pntd.0010059.s005.docx]

**S5Table. Relative abundance of mosquito species collected as larvae and pupae during routine house sampling in each locality and year.** Table shows the mean number per house (and total number collected) for each species detected and the confidence limits in brackets. All identified individuals were adults that had been reared in the laboratory from field-collected immature stages.

| Species | 1200 LG | | |  | Tabtenga | |  | Goundry | |
| --- | --- | --- | --- | --- | --- | --- | --- | --- | --- |
|  | 2016 | 2017 | |  | 2016 | 2017 |  | 2016 | 2017 |
| *Aedes aegypti* (pupae) | 1.06 (601)  [1.83-2.31] | | 2.44 (1,138)  [2.69-4.39] |  | 2.09 (1,492)  [2.59-3.69] | 3.64 (2,457)  [3.58-6.02] |  | 0.54 (579)  [1.37-1.75] | 1.14 (1994)  [1.74-2.62] |
|  | [1.83-2.31] | | [2.69-4.39] |  | [2.59-3.69] | [3.58-6.02] |  | [1.37-1.75] | [1.74-2.62] |
| *Aedes aegypti* (larvae) | 19.62 (9,317)  [17.31-24.56] | | 43.26 (11,373)  [34.03-57.56] |  | 27.96 (11,286)  [23.87-35.15] | 37.84 (14,550)  [30.15-50.05] |  | 3.36 (3,471)  [3.56-5.35] | 5.2 (5,420)  [4.77-8.05] |
|  | [17.31-24.56] | | [34.03-57.56] |  | [23.87-35.15] | [30.15-50.05] |  | [3.56-5.35] | [4.77-8.05] |
| *Aedes vittatus* (pupae) | (0) | | (0) |  | 0.01 (2) | 0.01 (3) |  | 0.47 (570) | 0.64 (1,006) |
|  | (0) | | (0) |  | [0.99-1.02] | [0.99-1.03] |  | [1.3-1.65] | [1.38-1.94] |
| *Aedes vittatus* (larvae) | 0.003 (1) | | 0.02 (269) |  | 0.02 (8) | 0.03 (15) |  | 1.93 (2,972) | 2.40 (2,878) |
|  | [0-1.00] | | [0-1.04] |  | [0.004.1.04] | [0-1.06] |  | [1.39-3.59)] | [1.68-4.30] |
| *Culex quinquefasciatus* (pupae) | 0.05 (248) | | 0.02 (3) |  | 0.16 (660) | 0.14 (163) |  | 0.04 (20) | 0.05 (24) |
|  | [1-1.1] | | [1-1.04] |  | [1.06-1.27] | [1.03-1.25] |  | [1.01-1.07] | [1.01-1.09] |
| *Culex quinquefasciatus* (larvae) | 0.4 (719) | | 0.48 (269) |  | 1.03 (1,241) | 0.48 (682) |  | 0.33 (275) | 0.47 (440) |
|  | [1.26-1.55] | | [1.26-1.73] |  | [1.73-2.38] | [1.27-1.74] |  | [1.21-1.46] | [1.29-1.68] |
| *Culex decens* | (154) | | (300) |  | (240) | (608) |  | (150) | (660) |
| *Culex nebulosus* | (142) | | (62) |  | (23) | (2) |  | (38) | (1899) |
| *Culex sp.* | (59) | | (4) |  | (5) | (13) |  | (197) | (2,892) |
| *Aedes hirsutus* | (0) | | (0) |  | (0) | (0) |  | (2) | (0) |
| *Aedes metallicus* | (0) | | (0) |  | (0) | (0) |  | (2) | (0) |
| Other *Aedes* | (0) | | (0) |  | (5) | (0) |  | (14) | (0) |
| *Anopheles gambiae s.l*. | (2) | | 0 |  | (21) | (4) |  | (6) | (4) |
| Other *Anopheles* | (1) | | (0) |  | (0) | (0) |  | (1) | (0) |
| *Lutzia tigripes* | (4) | | (1) |  | (11) | (4) |  | (534) | (520) |
