# Supplementary material for: First comprehensive analysis of Aedes aegypti bionomics during an arbovirus outbreak in west Africa: Dengue in Ouagadougou, Burkina Faso, 2016–2017
Source: PLoS Negl Trop Dis. 2022 Jul 6;16(7):e0010059. doi: 10.1371/journal.pntd.0010059 (PMC9321428; doi:10.1371/journal.pntd.0010059)
Supplement: S6 Table — (DOCX) [file pntd.0010059.s006.docx]

**S6Table**. Generalised linear mixed model of *Aedes aegypti* larval density. Reference factor levels of predictors are shown in square brackets, with beta effect size estimates, confidence intervals, z-value and probabilities for predictors included in the minimal model. Significant predictor terms are shown in bold and non-significant terms, not included in the model, are listed as a footnote.

| **Predictors** | Estimate | 95%CL | z-value | Pr(>\|z\|) |
| --- | --- | --- | --- | --- |
| **Intercept** | **2.88** | **[2.35-3.40]** | **10.72** | **<0.001** |
| Year [2016] |  |  |  |  |
| **2017** | **0.33** | **[0.10-0.57]** | **2.76** | **0.006** |
| Locality [Goundry] |  |  |  |  |
| **Tabtenga** | **0.33** | **[0.50-1.04]** | **2.76** | **0.006** |
| **1200LG** | **0.77** | **[0.34-0.90]** | **5.51** | **<0.001** |
| Month [August] |  |  |  |  |
| October | -0.18 | [-0.61-0.25] | -0.83 | 0.405 |
| **September** | **0.31** | **[0.05-0.56]** | **2.36** | **0.018** |
| 2-day rainfall | 0.01 | [0.00-0.02] | 1.72 | 0.086 |
| **Water level (cm)** | **0.02** | **[0.00-0.03]** | **2.30** | **0.022** |
| Container [Medium container] |  |  |  |  |
| Large container | 0.30 | [-0.01-0.61] | 1.90 | 0.057 |
| Others | 0.25 | [-0.72-1.23] | 0.51 | 0.610 |
| Small container | 0.18 | [-0.13-0.48] | 1.13 | 0.260 |
| **Car tire** | **0.35** | **[0.04-0.66]** | **2.21** | **0.027** |
| Animal drinking trough | 0.28 | [-0.09-0.66] | 1.48 | 0.140 |

**Non-significant terms**: temperature, container utility, container material, container height, water volume, number of residents, adult mosquito abundance, container position (shady/ sunny).
