# Supplementary material for: First comprehensive analysis of Aedes aegypti bionomics during an arbovirus outbreak in west Africa: Dengue in Ouagadougou, Burkina Faso, 2016–2017
Source: PLoS Negl Trop Dis. 2022 Jul 6;16(7):e0010059. doi: 10.1371/journal.pntd.0010059 (PMC9321428; doi:10.1371/journal.pntd.0010059)
Supplement: S7 Table — (DOCX) [file pntd.0010059.s007.docx]

**S7Table**. Generalised linear mixed model of *Aedes aegypti* pupal density. Reference factor levels of predictors are shown in square brackets, with beta effect size estimates, confidence intervals, z-value and probabilities for predictors included in the minimal model. Significant predictor terms are shown in bold and non-significant terms, not included in the model, are listed as a footnote.

| **Predictors** | Estimate | 95%CL | z-value | Pr(>\|z\|) |
| --- | --- | --- | --- | --- |
| **Intercept** | **6.01** | **[3.05-8.97]** | **3.98** | **<0.001** |
| Locality [Goundry] |  |  |  |  |
| **Tabtenga** | **0.51** | **[0.10-0.93]** | **2.41** | **0.016** |
| 1200LG | -0.18 | [-0.60-0.24] | -0.86 | 0.390 |
| **Temp mean** | **-0.14** | **[-0.25--0.04]** | **-2.72** | **0.007** |
| Container [Medium container] |  |  |  |  |
| Large container | -0.10 | [-0.59-0.39] | -0.41 | 0.684 |
| Others | 0.35 | [-1.15-1.86] | 0.46 | 0.646 |
| Small container | -0.17 | [-0.67-0.33] | -0.68 | 0.495 |
| Car tire | -0.17 | [-0.67-0.32] | -0.69 | 0.491 |
| **Animal drinking trough** | **0.71** | **[0.05-1.38]** | **2.12** | **0.034** |
| Container utility [No] |  |  |  |  |
| **Yes** | **-0.43** | **[-0.84--0.01]** | **-2.03** | **0.043** |
| **Adult abundance** | **0.02** | **[0.01-0.04]** | **2.54** | **0.011** |

**Non-significant terms**: Year, month, Temperature, container utility, container material, container height, water volume, water level, number of residents, cumulative rainfall of 7 previous days, container position (shady/sunny).
