# Supplementary material for: First comprehensive analysis of Aedes aegypti bionomics during an arbovirus outbreak in west Africa: Dengue in Ouagadougou, Burkina Faso, 2016–2017
Source: PLoS Negl Trop Dis. 2022 Jul 6;16(7):e0010059. doi: 10.1371/journal.pntd.0010059 (PMC9321428; doi:10.1371/journal.pntd.0010059)
Supplement: S1 Fig — (DOCX) [file pntd.0010059.s008.docx]

**S1Figure.** Mosquito community diversity in immature collections in each location and year.
