# Supplementary material for: First comprehensive analysis of Aedes aegypti bionomics during an arbovirus outbreak in west Africa: Dengue in Ouagadougou, Burkina Faso, 2016–2017
Source: PLoS Negl Trop Dis. 2022 Jul 6;16(7):e0010059. doi: 10.1371/journal.pntd.0010059 (PMC9321428; doi:10.1371/journal.pntd.0010059)
Supplement: Attachment — Submitted filename: Badolo et al review.docx [file pntd.0010059.s010.docx]

Badolo et al.: Bionomics of Aedes aegyptii during the 2016-2017 dengue outbreaks in Ouagadougou, Burkina Faso

Review report:

The manuscript by Badolo et al. characterizes the bionomics of the vector mosquito Aedes aegyptii in Burkina Faso in 2016 and 2017, and sampling coincided with dengue outbreaks in these years. Immature stages as well as adult mosquitos were collected and identified. In additions, the origin of the female bloodmeal of adult Aedes aegyptii females was determined using PCR. Findings include the characterization of a highly anthrophilic population with higher adult densities in urban than in rural location. Stegomyia indices proved less suitable indicators of adult populations, whilst other influences like container types and rainfall levels were stronger influences. The authors discuss their findings with respects to containment measures.

The present study is based on a large sample size and the analyses have been done with great care. Hence it provides a very useful resource to understand the bionomics of West African Aedes populations in relation to disease outbreak. It does not include data on the population during non-outbreak years or during the dry season, which could be included in future studies.

I recommend publication of the manuscript and I only have some minor comments on the text and figures, which are given below.

**Title**: The title seems to be underselling the manuscript a little bit. One could emphasize that this is the first comprehensive analysis of a West African population

**Abstract:**

L32: punctuation missing after PCR

L36: Isn’t Culex the most abundant mosquito sampled? See also my comments on table 1.

**Methods:**

L119 ff: How do the housing densities in the two urban areas compare?

**Results:**

L296 ff: Table1 indicates that Culex is the most abundant in the adult collections, which is opposing the statement in the Abstract that Aedes agyptii is the most abundant one, or does that only refer to immature stages, as indicated in figure S1? If Culex is most dominant in the adult samples but Aedes in the immature ones, how are these differences explained?

Also on table 1: I assume the fractions given for the Anopheles gambiae species indicate their percentage in the species complex and not in the overall sample. This should be indicated. But why do the numbers of Anopheles gambiae species do not add up?

L325 ff: Table 2: remove ‘§’ from headline. On right hand side some numbers seem to have shifted? Under % indoor the fraction of 1 rather than the percentage is given, which could be misunderstood at first glance.

L434 ff: Is there any idea/explanation why the overlap of immature and adult distribution is notably lower in the rural area? Why are adults completely missing from some sites where larvae were sampled?

L656 ff (Figure legends) The legends to figure 2 refers to figure 3 and vice versa, this needs to be corrected and the entire text should be checked if references to figures follow the right numbering

Also on figure legend 3 (referring to figure 2): The colour code is not given. In addition, the description ‘Proportion of positive containers for larvae’ does not fully fit what is shown. It seems to rather be a distribution of all larvae across the different container types, adding up to 100% in total

L667/ figure 4: I assume the dark black spots represent the houses that were sampled? This should be indicated in the figure legends

**Discussion:**

General: I would suggest the use of sub-headings in the discussion to improve structure and readability
