# Supplementary material for: First comprehensive analysis of Aedes aegypti bionomics during an arbovirus outbreak in west Africa: Dengue in Ouagadougou, Burkina Faso, 2016–2017
Source: PLoS Negl Trop Dis. 2022 Jul 6;16(7):e0010059. doi: 10.1371/journal.pntd.0010059 (PMC9321428; doi:10.1371/journal.pntd.0010059)
Supplement: Attachment — Submitted filename: Cover and Rebuttal Letter_PNTD-D-21-01724_R1_DW_AB.docx [file pntd.0010059.s011.docx]

Dear Editor,

We are pleased to submit the revision of our manuscript **PNTD-D-21-01724** “**First comprehensive analysis of Aedes aegypti bionomics during an arbovirus outbreak in west Africa: 2016-2017 dengue in Ouagadougou, Burkina Faso**” to *PLOS Neglected Tropical Diseases*  for publication*.*

We are grateful to the reviewers for their comments and have revised the manuscript accordingly, and provided point-by-point responses (with the reviewer queries in red). We have also made very minor corrections for typo errors and to improve English. We hope that our amendments will make this version suitable for publication.

Yours faithfully.

Philip P. McCall, MSc, PhD

Philip McCall

Professor of Medical Entomology

Vector Biology Department, Liverpool School of Tropical Medicine

Pembroke Place, Liverpool L3 5QA, UK

mccall@liverpool.ac.uk

Reviewer#1

We thank the reviewer for their helpful corrections throughout the manuscript all of which have been made as suggested.

Reviewer #2:

**Title:**

The title seems to be underselling the manuscript a little bit. One could emphasize that this is the first comprehensive analysis of a West African population

The title is changed to “First comprehensive analysis of Aedes aegypti bionomics during an arbovirus outbreak in west Africa: 2016-2017 dengue in Ouagadougou, Burkina Faso”.

**Abstract:**

(1) L32: punctuation missing after PCR.

The full stop has been added after PCR and the sentence has been modified to include parentheses as suggested by reviewer 1.

(2) L36: Isn’t Culex the most abundant mosquito sampled? See also my comments on table 1.

Culex were the most common in urban sites, and we have made a change to better illustrate the urban vs rural differences, focusing on *Aedes aegypti* and *Anopheles* spp.

**Methods**

(3) L119 ff: How do the housing densities in the two urban areas compare?.

Tabtenga was the locality with highest housing density compared to 1200LG, clarification is given in the text.

**Results**

(4) L296 ff: Table1 indicates that Culex is the most abundant in the adult collections, which is opposing the statement in the Abstract that Aedes aegypti is the most abundant one, or does that only refer to immature stages, as indicated in figure S1? If Culex is most dominant in the adult samples but Aedes in the immature ones, how are these differences explained?

The abstract has been corrected as described above; this refers to adult samples

Also on table 1: I assume the fractions given for the Anopheles gambiae species indicate their percentage in the species complex and not in the overall sample. This should be indicated. But why do the numbers of Anopheles gambiae species do not add up?

For *Anopheles gambiae* species, not all the collected mosquitoes have been subjected to PCR identification, explaining why the numbers don’t sum to the *An. gambiae sl*. total. This is indicated in Table 1 legend by addition of ‘The proportions of *Anopheles gambiae* complex members were calculated using the total number of *An. gambiae s.l.* identified to species (rather than total collected) as denominator.’ In the meantime, we increased the sample size for identified *Anopheles gambiae* complex mosquitoes to species in Table 1.

(5) L325 ff: Table 2: remove ‘§’ from headline. On right hand side some numbers seem to have shifted? Under % indoor the fraction of 1 rather than the percentage is given, which could be misunderstood at first glance.

The symbol is removed from headline. The numbers are expressed as percentages now and we made sure that the numbers have not shifted.

(6) L434 ff: Is there any idea/explanation why the overlap of immature and adult distribution is notably lower in the rural area? Why are adults completely missing from some sites where larvae were sampled?.

We have added the following possible explanation in the discussion ‘The lower overlap in adult vs. larval densities in the rural compared to urban locations might be explained by a closer proximity of vegetation around households in the former, which offer potentially suitable, but difficult to sample, resting places for adults.

(7) L656 ff (Figure legends) The legends to figure 2 refers to figure 3 and vice versa, this needs to be corrected and the entire text should be checked if references to figures follow the right numbering. Also on figure legend 3 (referring to figure 2): The colour code is not given. In addition, the description ‘Proportion of positive containers for larvae’ does not fully fit what is shown. It seems to rather be a distribution of all larvae across the different container types, adding up to 100% in total

The legends have been checked and now refer to the appropriate figures and the color codes are given in figure 3 and the title has been changed to correspond to the figure.

L667/ figure 4: I assume the dark black spots represent the houses that were sampled? This should be indicated in the figure legends

This is correct and has been added to Fig 4 legend

**Discussion:**
(7) General: I would suggest the use of sub-headings in the discussion to improve structure and readability

Sub-headings are now added to the discussion
